# Recurrent Goiter Presented with Marine-Lenhart Syndrome 27 Years After Initial Surgery

**DOI:** 10.7759/cureus.5768

**Published:** 2019-09-26

**Authors:** Emin Gurleyik

**Affiliations:** 1 Surgery, Duzce University Medical Faculty, Duzce, TUR

**Keywords:** thyroid, hyperthyroidism, graves' disease, adenoma, nodule

## Abstract

Marine-Lenhart syndrome (MLS), a rare form of hyperthyroidism, is the coexistence of Graves’ disease (GD) and autonomously functioning thyroid nodule(s). Herein, we report a case of recurrent goiter presented with MLS. A 52-year-old man presented at our department with recurrent goiter, exophthalmia, and symptoms of hyperthyroidism. In addition to clinical signs and thyroid eye disease, suppressed thyroid-stimulating hormone (TSH) and high free thyroxine (FT4) and autoantibody levels lead to the diagnosis of GD. Thyroid ultrasound and nuclear scan showed the presence of a large, solid, and functioning “hot” nodule in the right lobe. Thus, in recurrent goiter cases, the diagnosis was MLS, wherein autoimmune hyperthyroidism was associated with the functioning nodule. Following medical control with methimazole, the patient underwent total excision of recurrent goiter. Levothyroxine (LT4) therapy was prescribed to maintain normal serum hormone levels. At follow-up, the gradual decrease in serum levels of autoantibody was detected. This patient is a very rare example of MLS that occurs in recurrent goiter case. Clinical signs, serum hormone and autoantibody levels, thyroid ultrasound, and nuclear scan establish the correct diagnosis of this specific and rare disorder. Thyroid surgery and total removal of glandular tissue provides definitive control of hyperthyroidism and obviates autoimmune reaction.

## Introduction

Marine-Lenhart syndrome (MLS) is described as the coexistence of Graves’ disease (GD) and autonomously functioning thyroid nodule(s), both of which cause hyperthyroidism by different physiopathology [1-2]. In toxic adenoma and toxic multinodular cases, solitary or multiple hyperactive nodules may create thyrotoxicosis, while in GD, hyperthyroidism arises from autoimmune diffuse hyperplasia of the thyroid gland. GD is an autoimmune disorder caused by an autoantibody that stimulates thyrotropin receptors of follicular cells, leading to the excessive synthesis of thyroid hormones. In patients with GD, thyroid glands may harbor solid nodules, of which, the great majority are hypofunctioning. In general, thyroid nodules occur in 10% to 35% of GD cases [1-3]. Uncommonly, these nodules are hyperfunctioning adenomas that are estimated to occur in 0.8% to 2.7% of GD cases. The syndrome of exophthalmic goiter with incidental functioning nodule(s) was recognized as a distinct subdivision of GD. In comparison with toxic multiple nodules, the association of GD with solitary toxic adenoma is much rarer [4]. During the literature search, we could not find a case of MLS in a patient with recurrent goiter. In this report, we describe a rare case of recurrent goiter presenting with MLS that occurred many years after the initial surgery.

## Case presentation

A 52-year-old man presented to our department with symptoms of hyperthyroidism including asymmetrical anterior neck mass, palpitation, weight loss, and heat intolerance. He had undergone thyroid surgery, probably subtotal resection, 27 years ago. Physical examination, inspection, and palpation showed marked exophthalmia and nodular enlargement of the thyroid remnant, which is more apparent at the right side. Blood chemistry and hormone analysis confirmed thyrotoxicosis, while the presence of elevated serum thyroid peroxidase autoantibody (anti-TPO) and thyrotropin receptor autoantibody (TRab) levels showed an autoimmune basis of thyroid disorder. Exophthalmia, increased free thyroxin (FT4) and autoantibody, and suppressed TSH levels lead to the diagnosis of GD.

**Figure 1:**
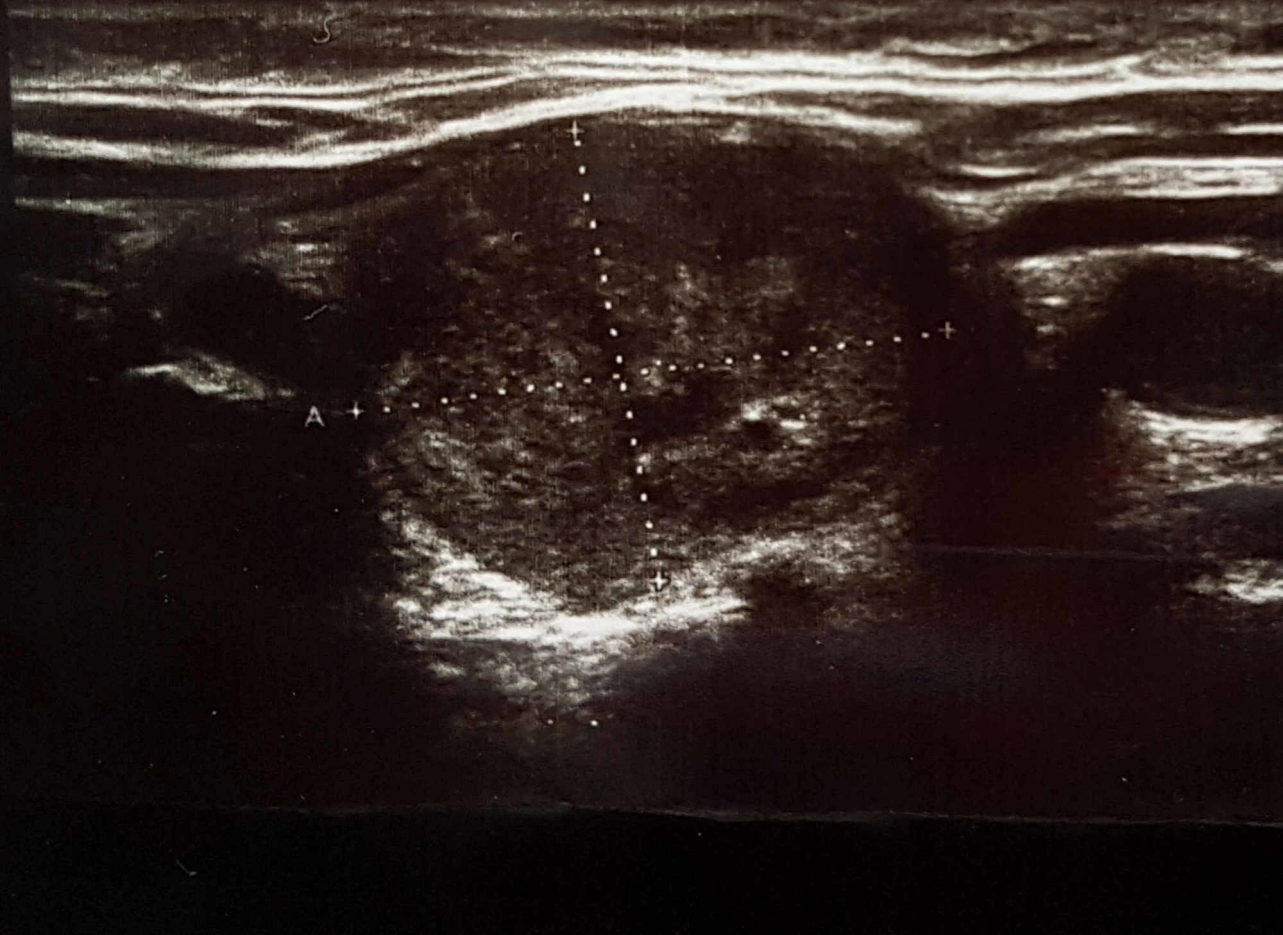
Right lobe ultrasound Ultrasound shows a heterogeneous solid nodule that is a functioning "hot" nodule in thyroid scintigraphy.

Thyroid ultrasound revealed the dimensions of the recurrent goiter mass: right lobe remnant, 24 × 31 × 47 mm and left lobe remnant, 20 × 30 × 46 mm. Meanwhile, 27 × 20 × 30-mm and 27 × 20 × 25-mm heterogeneous nodules in the right and left lobes, respectively, were detected as well (Figure 1).

**Figure 2:**
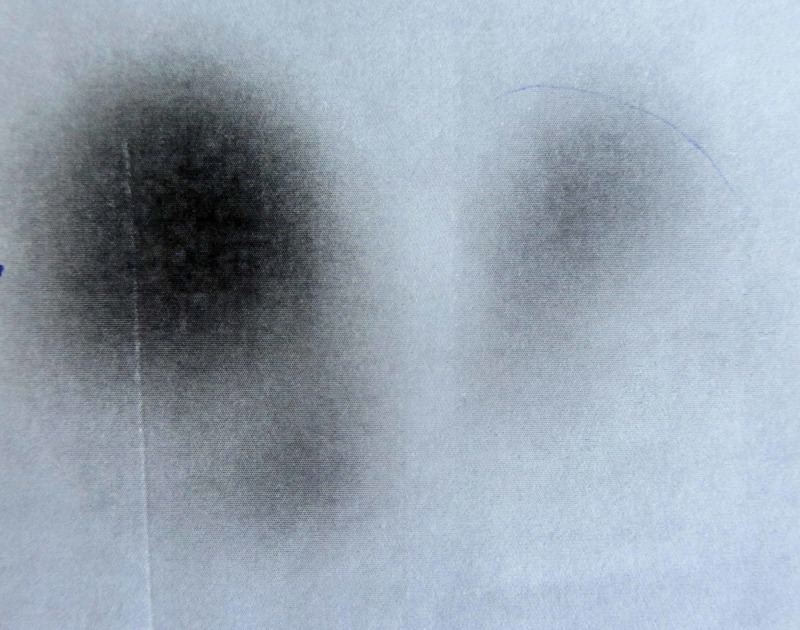
Nuclear scan of the thyroid Thyroid scintigraphy shows functioning "hot" nodule, adenoma in the right lobe.

Thyroid scintigraphy of the gland showed a large autonomous hyperactive functioning nodule in the right lobe and normal or hypoactive nodule in the left lobe (Figure 2).

Moreover, fine-needle aspiration from nodules showed benign cytology.

**Figure 3:**
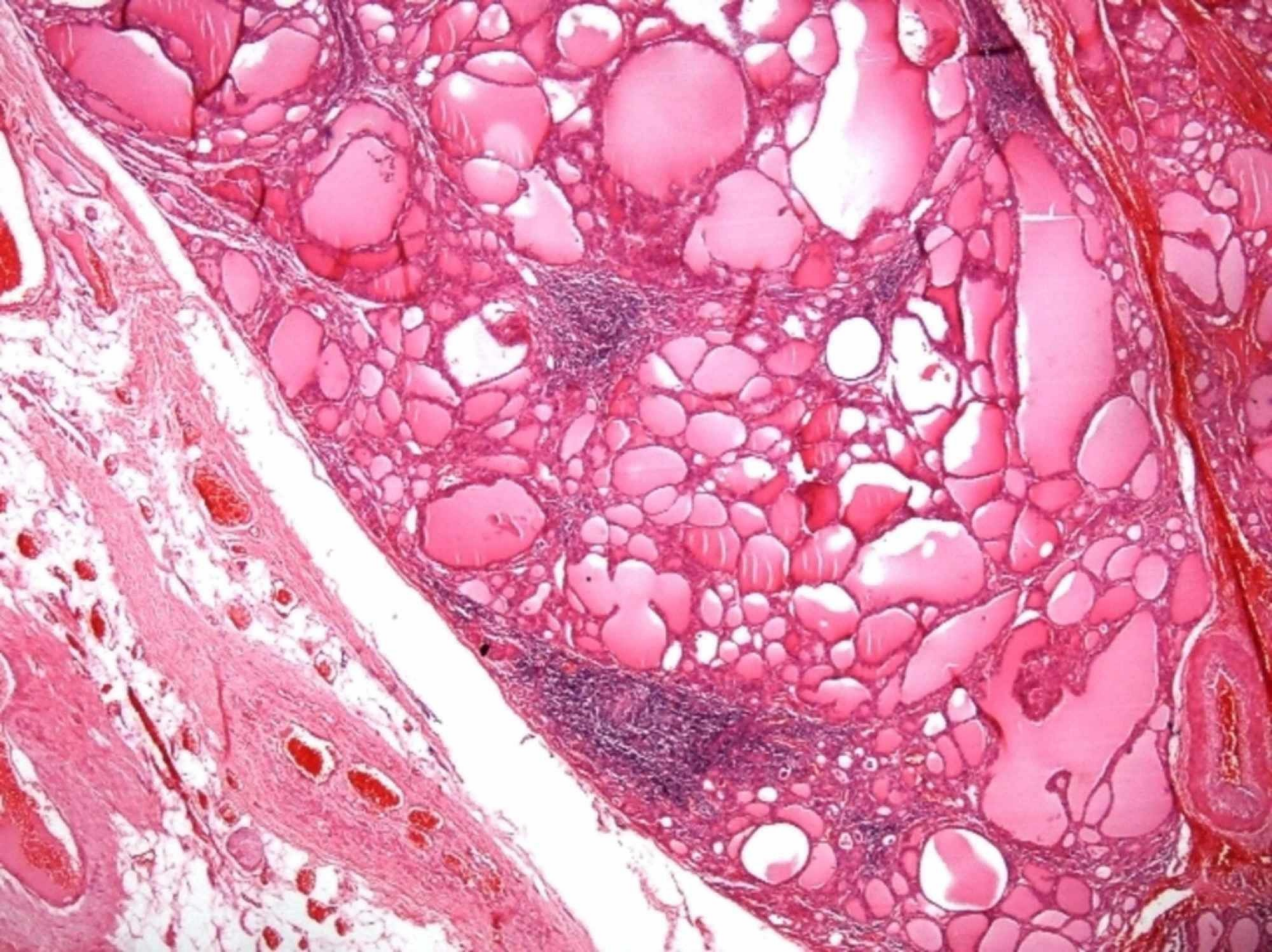
Pathologic photomicrograph Nodular goiter and lymphocytic thyroiditis

We observed and found symptoms and signs of hyperthyroidism, exophthalmia, and autoimmune thyrotoxicosis with high autoantibody levels, associated with hyperactive functioning adenoma. Thus, the diagnosis of MLS was made. After normalizing thyroid function through the prescribed antithyroid medication (methimazole), the patient underwent total thyroidectomy for the removal of recurrent goiter mass. Then, the patient was discharged on the second postoperative day. Thereafter, normal serum FT4 level was maintained with levothyroxine (LT4) therapy at a dosage of 125 µg/day. Accordingly, the histopathologic analysis showed follicular adenoma in the right lobe, multinodular goiter, extranodular diffuse hyperplasia, and lymphocytic thyroiditis (Figure 3).

Consequently, serum TSH and FT4 levels were normal, and serum autoantibody levels gradually decreased in the first and third months and one year postoperatively (Table 1). In the follow-up, the patient stated relief of thyroid eye disease secondary to exophthalmia.

**Table 1:** Results of biochemical analysis at preoperative period and postoperative follow-up TSH, thyroid-stimulating hormone; FT4, free thyroxin; anti-TPO, anti-thyroid peroxidase autoantibody; TRab, thyrotropin receptor autoantibody; anti-TgAb, anti-thyroglobulin antibody; LT4, levothyroxine

	TSH	FT4	Anti-TPO	TRab	Anti-TgAb	Thyroglobulin
Reference value	0.4-4 uIU/mL	0.6-1.12 ng/dL	0-60 IU/mL	0-9 IU/L	0-40 IU/mL	1.6-60 ng/mL
Preoperative before methimazole	<0.005	3.48	>1300	20	0.1	86.4
Preoperative after methimazole	0.07	1.09	976		0.1	46.8
Surgery	Total thyroidectomy 125 µg/day LT4					
Postoperative first month	0.763	0.95	278.5	11.38	0.1	0.2
Postoperative third month	1.029	0.82	42.7	5.7	0.1	0.1
Postoperative one year	0.443	1.21	24.1	3.8	0.1	0.2

## Discussion

Etiology of hyperthyroidism is mainly based on two different pathogeneses: nodular or diffuse hyperactivity of the gland. In GD, autoimmune-based disorder of the thyroid induces diffuse hyperplasia increasing hormone production and secretion, while in toxic nodular goiter, autonomously functioning nodules produce and secrete excessive thyroid hormone. Uncommonly, since both pathogeneses occur and coexist in the same case, this disorder is called MLS. Therefore, the term MLS describes the association between GD and functioning thyroid nodule(s), such as toxic adenoma and toxic multinodular goiter. While Charkes has reported the prevalence of this disorder at approximately 2.7% based on a review of 375 GD cases, Carnell and Valente detected autonomous nodules in only four (0.85%) of 468 GD cases [4-5]. This condition is estimated to occur in 0.8% to 2.7% of patients with GD; also, thyroid nodules occur in 10% to 15% and functioning nodules occur in 1% of patients with GD [2,4-7]. In the literature, MLS was not previously reported in a recurrent goiter case. Our present patient with recurrent goiter is a very rare example of MLS that occurred many years after the initial surgery. In our case, serum hormone levels confirm the diagnosis of hyperthyroidism, although high autoantibody levels also support the autoimmune basis of the disorder. Additionally, the coexistence of a large functioning “hot” nodule contributes to the occurrence of thyrotoxicosis. However, Lombardi et al. have reported symptoms of hyperthyroidism wherein physical and clinical signs and biochemical results (thyroid hormones and autoantibodies) were consistent with GD (autoimmune hyperthyroidism) associated with an autonomously hyperactive nodule, suggesting an MLS diagnosis. Likewise, our patient presented with symptoms of hyperthyroidism associated with marked exophthalmia [1].

In addition to thyrotoxic symptoms, some clinical, laboratory, imaging, and pathological signs and results were suggested for the correct diagnosis of MLS [2]. Our patient presented with symptoms of hyperthyroidism associated with marked exophthalmia. Some further steps of work-up lead to the final diagnosis of MLS.

1. Thyroid function tests consistent with hyperthyroidism: Biochemical serum analysis of our patient showed suppressed TSH and high FT4 levels, which are laboratory findings of hyperthyroidism.

2. Inclusive of serological testing for GD: High serum levels of both anti-TPO and TRab show an autoimmune basis of the hyperthyroidism in our patient.

3. Thyroid nodularity by ultrasound

4. The presence of “hot” nodule(s) by nuclear scan: After the ultrasound and nuclear scan, we can comment that in our patient with autoimmune hyperthyroidism, an ultrasound showed the presence of solid nodules, in which hyperfunction was proved by a hot nodule in the nuclear scan.

5. Biopsy showing follicular adenoma, diffuse hyperplasia of follicular cells, and lymphocytic infiltration [8] Hence, the histopathology report of our patient also supports the diagnosis of MLS.

Total resection of recurrent goiter resolved hyperthyroidism in the present case, and normal hormone levels have been maintained with the prescription of LT4. In the patient with MLS, total thyroidectomy is the surgical intervention of choice which provides radical treatment and definitive control of thyrotoxicosis. In the follow-up of our patient, the decrease in serum levels of autoantibody showed gradual alleviation of the autoimmune reaction. Aside from the radical treatment of hyperthyroidism, the total removal of glandular tissue also obviates the autoimmune reaction created by GD. Of great significance, relief of autoimmune response is especially helpful to overcome thyroid eye disease due to severe exophthalmia. Malignancies such as papillary cancer have also been reported in patients with MLS [1,9-10]. Generally, total thyroidectomy is the recommended procedure for surgical management of MLS [1-2,8-10].

## Conclusions

MLS is a rare form of hyperthyroidism that may occur in the remnant tissue causing recurrent goiter many years after the initial surgery. In the patient with clinical symptoms and signs, as well as with exophthalmia, hormone analysis establishes the diagnosis of hyperthyroidism. The serum levels of autoantibody reveal the autoimmune basis of the disorder (GD). Further, ultrasound and nuclear scan of the thyroid detect the presence of solid and hot functioning nodule(s) contributing to hyperthyroidism that confirms the association of both causes of thyrotoxicosis in the same patient. Furthermore, the total removal of glandular tissue provides definitive control of hyperthyroidism and relief of autoimmune reaction.
